# Monkeys do not show sex differences in toy preferences through their individual choices

**DOI:** 10.1186/s13293-023-00489-9

**Published:** 2023-02-03

**Authors:** Florent Pittet, Victoria Heng, Jala Atufa, Eliza Bliss-Moreau

**Affiliations:** 1 Neuroscience and Behavior Unit, California National Primate Research Center, University of California, County Road 98 at Hutchison Drive, Davis, CA 95616 USA; 2 Department of Psychology, University of California, Davis, USA

**Keywords:** Gender socialization, Macaca mulatta, Nonhuman primates, Object play, Sex differences, Social development

## Abstract

**Background:**

As interest in evaluating sex differences in nonhuman animals grows, the finding that male and female monkeys have toy preferences that differ, and that parallel those documented in human children, has garnered significant attention and is leveraged as an argument in favor of a biological contribution for human sex differences. To date, however, only two studies have investigated sex differences in monkeys’ toy preferences, both documenting that males prefer toys considered to be “masculine” (such as vehicles) and females prefer toys considered to be “feminine” (such as dolls). Monkeys in these studies were tested in their social groups, making it hard to determine if the sex differences reported reflect actual individual preferences or result from social dynamics present at the time of testing.

**Method:**

Here, we assessed the preferences of 14 rhesus macaques (*N* = 7 males; *N* = 7 females) who were singly tested in a choice test with a variety of toys characterized as masculine (hard non-zoomorphic wheeled toys), feminine (zoomorphic soft toys), neutral (hard non-zoomorphic toys) and ambiguous (zoomorphic or plush vehicles) based on criteria from previous studies.

**Results:**

Males and females showed similar preferences for neutral and “masculine” toys and preferred them (i.e., were more likely to interact with them) to “feminine” and sex-ambiguous toys. When they interacted with the toys, both males and females interacted more with neutral than with “masculine” toys. Females, but not males, interacted more with neutral and “masculine” toys than with “feminine” toys. The highest frequency of interaction for any single toy for the male monkeys was with the doll—standing is stark contrast to previous findings.

**Conclusions:**

Our results contrast greatly with the previous study in rhesus monkeys, as well as findings in human children, suggesting that the previously documented sex differences are likely context dependent, and question the existence of a strong biological basis to sex differences in toy preferences.

**Supplementary Information:**

The online version contains supplementary material available at 10.1186/s13293-023-00489-9.

## Highlights

- When tested outside of their social groups, male and female rhesus macaques show no sex differences in their toy preferences.
- Males and females were both more likely to interact with neutral toys and “masculine” toys than with “feminine” toys and sex-ambiguous toys.
- When they interacted with toys, males interacted more with neutral toys than with “masculine” toys.
- When they interacted with the toys, females interacted more with neutral toys than with “masculine”, “feminine” or ambiguous toys.
- Only one between-sex difference emerged: when they interacted with it, males interacted more with the doll more than females did.

**Supplementary Information:**

The online version contains supplementary material available at 10.1186/s13293-023-00489-9.

## Introduction

After many decades of ignoring sex differences in animal models, their study is now increasingly taking center stage [1], in large part because of the US National Institutes of Health mandate to evaluate sex as a biological variable [2]. In behavioral neuroscience evaluating both sexes is requiring nothing short of a major cultural revolution [3], although it is mostly limited to studies of rodents, as nonhuman primate research is largely exempt. Despite being exempt for sex as a biological variable requirement, studies of sex differences in nonhuman primates (NHP) are incredibly important. Research conducted in NHPs serves as a critical translational bridge between in vitro and other animal model studies, and human interventions because of their biological and behavioral similarities to humans [4]. Historically, in most domains of NHP science, males are used as subjects almost exclusively, making the translatability of results questionable, particularly when there are well documented sex differences in the observed prevalence of multiple psychiatric disorders in humans [5], neurodevelopmental [6], and neurodegenerative [7]. Trying to understand the mechanisms that support the emergence of sex differences in people is challenging due to the complex interplay of biological, psychological, and social factors [8] and inability to ethically causally manipulate those factors in people. NHP models allow for such manipulations and the nascent similarities that exist between NHPs and humans speak to potentially shared evolutionary mechanisms. Yet, little research specifically addressing sex differences in nonhuman primates exist.

In the nonhuman primate psychological literature, one sex difference that has captured a significant amount of attention is the finding that male and female monkeys have different preferences for different types of toy objects [9, 10]. These findings are particularly enticing because they mirror those believed to exist in human children [11]. Further, tasks that ask monkeys to interact with objects, like toys, are used in a wide variety of studies to evaluate everything from personality (e.g., [12, 13] to affective processing (e.g., [14] to the function of particular brain regions or neural networks in behavior (e.g., [15–18].

In the human literature, the idea that male and female children^1^ prefer different types of toys is widespread, is thought to speak to gender socialization and is also leveraged in some theories of autism and neurodevelopmental disorders [20, 21]. Very early work demonstrated that boys had strong preferences for “masculine” toys and girls showed weaker preferences for “feminine” toys [22]. In that early report, the gendered categories to which toys were assigned were based on assumptions by researchers, but more recent studies assign gendered categories based on adult reports of the masculine or feminine nature of the toy (which also relies on assumptions/stereotypes, but at least surveys a broader sample than the researchers alone) [23]. There is now a sufficiently large literature on people’s sex differences in toy preferences that meta-analyses could be carried out, they do confirm the existence of these sex differences in toy preference in people [11, 23]. Young girls show more interest than boys do in toys rated as feminine (typically dolls) and boys show more interest than do girls in toys rated as masculine (typically vehicles). These toy preferences could be socially influenced to correspond to socialized stereotypes about gender [24], although there is some evidence that the origin of these preferences may have a biological component as well.

The ontogeny of sex differences in toy preferences has been at the heart of multiple theories relating to such biological (focusing mostly on hormonal action) and environmental (focusing mostly on gender socialization) origins of sex differences. Hormonal theories of gender development suggest that the organizational action of prenatal androgens during critical periods of early development shape variation in sensory, cognitive, and social behavior which support sex differences in interest in objects [25]. Multiple studies in animal models have demonstrated that artificial increases in androgen levels during prenatal (primates) or early postnatal (rodents) development of females induce a long-lasting masculinization of their socio-sexual behavior [26–28]. In humans, genetic girls with congenital adrenal hyperplasia (CAH) are exposed to increased testosterone levels prenatally and display, postnatally, enhanced male-typical behaviors, including toy preferences similar to those expressed by boys [29, 30]. Studying genetic girls with CAH who have been socialized like typical genetic females (e.g., parents encourage sex-typical toy play, [29]) provides a rare opportunity to observe male typical toy preferences in genetic girls thought to emerge because of the variation in their hormone profiles. Sex differences in toy preferences are also apparent in visual preferences for dolls and trucks respectively, by young girls and boys, before the age when they themselves theoretically should be able to conceptualize masculine and feminine categories [31].

In contrast to hypotheses about hormonal action, hypotheses about environment and gender socialization suggest that sex differences in toy preferences emerge primarily from the action of social reinforcers related to concepts about gender identity [31]. This view suggests that sex-specific behavior emerges when concepts about gender identity are socially constructed or built by interactions with other people. For instance, despite self-reports that they treated their sons and daughters similarly, parents were more engaged and provided more positive feedback to their sons and daughters for playing with sex-typical toys and more discouragements for playing with a cross-sex toys [32–34]. At 4 years old, preschoolers are already aware of social expectations of genders and adjust their toy preferences accordingly [35].

Understanding the relative and interactive roles of biological and social contributions on the development of toy preferences in children is challenging because, for social species, biological and social features are entirely intermingled and the dichotomy between them is well regarded as being simply false [36–38]. Controlling for, or manipulating, biological and social developmental parameters is neither realistic nor ethical in people, and children begin to gender self-identify very early and experience sex-biased interactions from parents and other peers, making it particularly complex to investigate causality [10]. An alternative approach is to look at sex differences in toy preferences in relevant nonhuman models. Not only does it offer the experimental flexibility to control or manipulate the hormonal signaling during critical periods of development (e.g., [39]) or social environment [40] but also have the potential to bring new critical insight into the evolutionary roots of sex differences and their mechanisms in humans [41]. In this vein, two studies to date have investigated sex differences in toy preferences in nonhuman primates—one in vervets (*Cercopithecus aethiops sabaeus* [9] and one in rhesus macaques (*Macaca mulatta* [10]).

Despite discrepancies in methods and results (see [42] for details), the two studies evaluating sex differences in toy preferences in monkeys [9, 10] showed striking similarities with results gleaned from human children. Vervet males spent more time than vervet females in contact with toys typically preferred by human boys (e.g., police car or orange ball), and vervet females spent more time than vervet males in contact with toys typically preferred by human girls (e.g., a doll or a red pan). Vervet females spent more time in contact of toys typically preferred by human girls than in contact with toys typically preferred by human boys, but vervet males did not prefer one type of toy over the other [9]. The subsequent study in rhesus macaques [10] showed that female rhesus monkeys interacted more with toys associated with human girls (plush toys) than did rhesus males, but rhesus males and females did not differ in their interactions with toys associated with human boys (wheeled toys). Additionally, rhesus males interacted more with human boy toys than with human girl toys while rhesus females did not show any significant preference.

The evidence that there are sex differences in toy preferences in two monkey species [9, 10] that parallel what has been observed in human children [11, 23] has been leveraged as an argument in favor of biological contribution for sex differences in toys preferences (e.g., [11]. These are strong conclusions to be drawn from relatively scant evidence, and further evidence gleaned from experimental contexts with significant limitations. For example, Alexander and Hines [9] presented the toys singly and sequentially which does not allow for a proper measurement of individual preferences because the animals were not put in a position where they had to make a choice between objects to demonstrate a preference for one compared to the other [43]. Additionally, in both studies, the authors made a very small number of toys available to entire social groups: Alexander and Hines [9] presented one toy at a time to groups ranging from 17 to 28 monkeys (total of 6 toys tested) and Hassett et al. [10] presented two toys at a time to a group of 135 monkeys (total of 13 toys tested). These group contexts make it difficult to evaluate individual preferences as such, because the ability and motivation of an individual monkey to approach and interact with each stimulus depends on the actions of the other group members. For instance, Hassett et al., [10] report that the number of interactions with the toys depended on females’ but not males’ dominance rank suggesting that an individual’s access to the toys was dependent on social context. This is particularly important considering that more than half of their sample was excluded from the analysis because they did not interact with the toys, possibly leading to a focus on a biased subsample of their population. Similarly, in the vervet study, Alexander and Hines [9] reported a trend for higher ranking male vervets to interact more with masculine and less with feminine toys while dominance had no effect in females. In this same study, the authors reported that the inclusion of dominance rank produced results “essentially the same”, but the authors do not report controlling for an interaction between dominance and sex, critical if hierarchy influences access to toys differently in males and females. In addition to dominance, multiple other social mechanisms such as social facilitation or stimulus enhancement or competition are likely to operate differently in males and females and so the methodical choice to test animals in groups influence the conclusions drawn from these two studies.

If sex differences in toy preferences are tested by observing individuals in a social group, one cannot dissociate intrinsic individual preferences from socially driven preferences. This is critically important when the interdisciplinary effort aims at understanding the relative and interactive contributions of biological and social parameters in the expression of sex differences. If sex differences in toy preferences in primates rely at least partially on biological mechanisms, then they should emerge through individual preferences, outside the social context, a phenomenon that can be tested by observing monkey’s preferences when tested solo in choice tests.

Here, we evaluated toy preferences of 14 adult rhesus macaques (7 males and 7 females) tested alone in a choice test. The macaques were socially reared to adulthood in large outdoor groups and then housed with a compatible social partner indoors. With particular attention to reproducibility, we used a set of toys modeled as closely as possible to those previously tested in rhesus macaques by Hassett et al. [10] where “masculine toys” are wheeled toys and “feminine toys” are plush toys with animal features (hereafter zoomorphic). Additionally, we included two new categories of toys: toys that are neither wheeled nor zoomorphic (which we call “neutral”) and toys that are both wheeled and plush or wheeled and zoomorphic (which we call “ambiguous”). Unlike previous studies, subjects were tested alone. Preferences were tested in a choice-paradigm that allowed animals to interact with only one of two toys at a time; the likelihood to interact and the number of interactions were computed for each toy within each trial as an index of preference. If it was the case that the effects of previous studies were not dependent upon the social context of their evaluation, then we reasoned we should see comparable sex differences to what was previously observed. If it was the case that social context is at least partially responsible for the effects previously reported, then we reasoned we would not see such sex differences.

## Materials and methods

All protocols were approved by the University of California Davis Institutional Animal Care and Use Committee and carried out in accordance with the US National Institutes of Health guidelines. All procedures were performed at the California National Primate Research Center (CNPRC).

### Subjects and housing conditions

Subjects were *N* = 14 adult (7 females and 7 males, median age: 12.65, range 9–14) rhesus macaques (*Macaca mulatta*), born at the CNPRC and raised by their mothers in large outdoor multi-male multi-female groups. At the time of enrollment for the experiment, monkeys underwent physical examinations prior to check for the absence of health problems and normal sensorial capabilities. During the experiment, all the adult animals were socially housed in mixed-sex pairs with a compatible partner, each having access to a standard adult macaque laboratory cage (66 cm wide × 61 cm long × 81 cm high) and their partner’s cage. They were either paired continuously or for 6 h per day according to their ability to share food.

The housing room was maintained on a 12:12 LD cycle with lights on at 0600. Monkeys were fed monkey chow (Lab Diet #5047, PMI Nutrition International INC, Brentwood, MO) twice daily and provided with fresh fruits and vegetables twice per week. Water was accessible ad libitum. Animals additionally received food enrichment (daily cereal mix delivery on their forage boards, periodic delivery of fruits and vegetables in puzzle balls and puzzle tubes, monthly provision of fresh coconuts) and daily access to a toy (Kong rubber toy, Nylabone chew toys, or metal balls).

### Stimuli

**Fig. 1:**
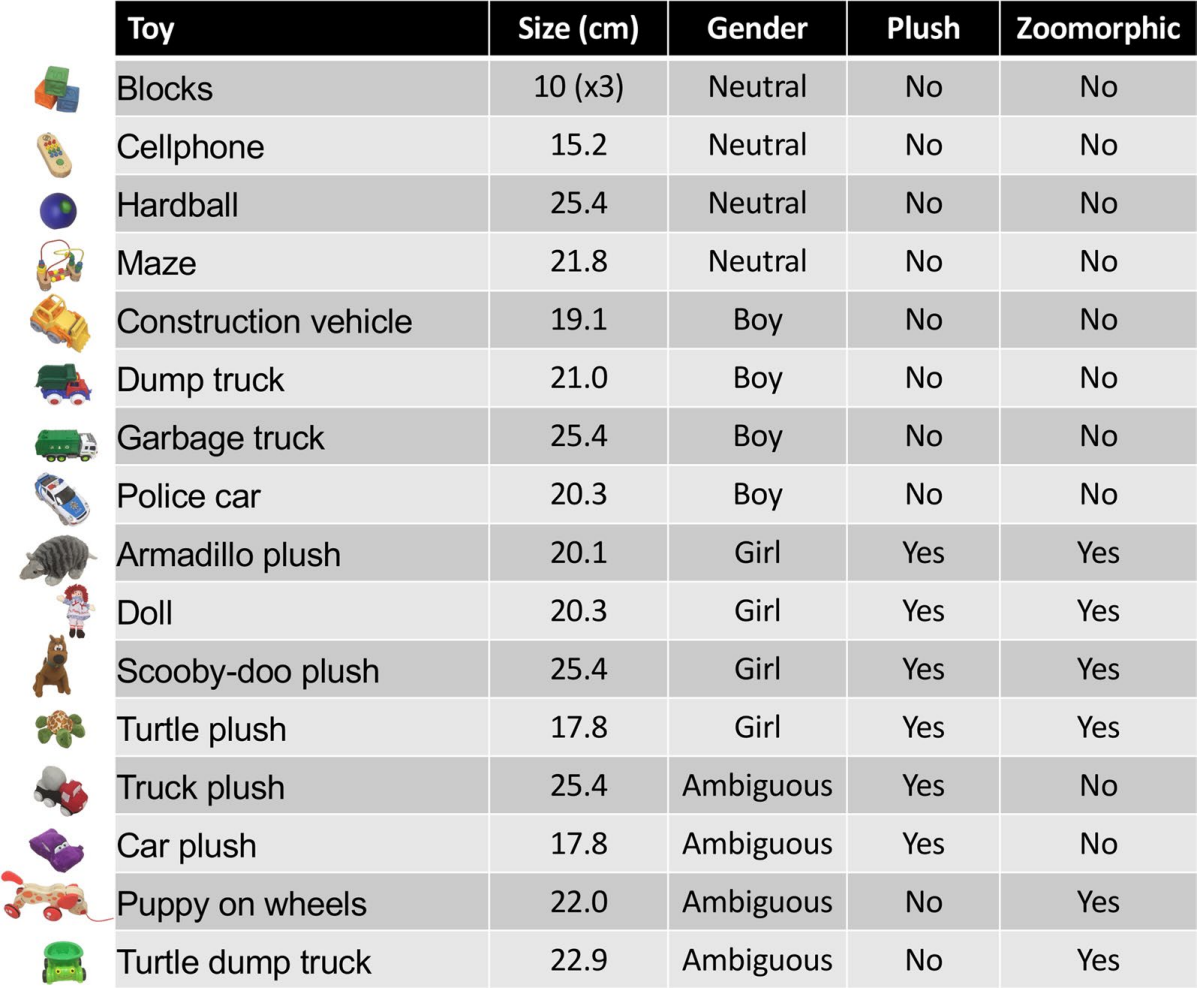
List of toys used in the study

Preferences for toys were tested using a choice test paradigm between April and June 2017. The stimuli consisted in a collection of 16 toys (median size: 20.66, range 10–25.4 cm) in this study: masculine and feminine toys replicating Hassett et al. [10], as well as neutral toys (not having features of either masculine or feminine toys and ambiguous toys (having features of both masculine and feminine toys) (See Fig. 1). Masculine toys or wheeled toys (made of hard materials and have moving parts): Construction vehicle, Dump truck, Garbage truck, Police car, and. Feminine toys or plush toys (soft plush or fabric toys that resemble animals / have clear faces): Plush armadillo, Raggedy-Ann doll, Plush Scooby-Doo and Plush turtle. Neutral toys (not plush and not zoomorphic): Squeezable stacking blocks, Wooden cell phone, Hard ball, Wooden maze. Ambiguous toys (have features of both masculine toys and feminine toys, either hard material and zoomorphic vehicle or plush vehicle): Cement truck plush, Car plush, Wooden puppy on wheels, Plastic turtle dump truck.

### Apparatus and behavioral test

Monkeys were tested for their toy preferences using a choice test paradigm. The apparatus consisted of three adjoined standard nonhuman primate cages of equal dimensions (66 wide × 61 long × 77.5 high cm). Monkeys were placed in the center cage at the start of each trial and toys were placed in the side cages. Both side cages contained an inner mesh wire compartment (46 wide × 38 long × 54 high cm) where the stimuli were placed. The inner compartment was attached to the front wall of the larger cage. Cages were separated by sliding doors fitted with small mesh windows, allowing the animal to see the stimulus in the side cages from the central cage when closed, and to freely move to the side cages when opened.

Monkeys were acclimated for 3 days and then tested for 12 days between 0900 and 1600. For both acclimation and testing, they were moved from their home room to the testing room and transferred to the central cage. Each individual was moved and tested alone. Once in the central cage, the monkey was left undisturbed for one minute, before the sliding doors were opened, and the monkey was free to move between the three compartments for 5 min. When the trial was over, the monkey returned to the central cage, the doors were closed, and the food reward (acclimation phase) or the toys (testing phase) were replaced for the following trial. Between each trial, the monkey was left undisturbed for one minute before the items were made accessible.

Monkeys first underwent an acclimation phase with three trials per day for at least 4 days. A food item was placed in each side cage and the animals accessed the testing phase only if they accessed both side cages and ate the food item for 10 trials on 2 consecutive days. All the monkeys (*N* = 14) validated this phase and accessed the testing phase.

During the test phase, monkeys were tested with the toys. Each monkey was tested for all the 120 possible toy pairs (10 consecutive trials daily for 12 days). All monkeys were exposed to the same randomized sequence of pairs of toys across these trials. When the monkeys were done with their 10 trials (on a given test day), they were transported back to their home cage and the apparatus was fully cleaned before the following monkey was tested.

Trials were videotaped. From these videos, the observer extracted: the time spent interacting with each toy and all interactions with each toy. Interactions included hand interactions (pulling, pinching, poking), mouth interactions (licking, biting), and sniffing. The observer also noted the presence stereotypies and the frequencies of coos, barks, cage shakes and scratches.

### Data analysis

The analyses were computed using R 4.1.1 [44] with the packages lme4 [45], car [46], fitdistrplus [47] and emmeans [48].

We first looked at sex-related differences in the likelihood to interact with the different toys and the number of interactions with the different toys when the animal interacted with a given toy at least once. The duration of interaction with the different toys was strongly correlated with the number of interactions (*r* = 0.95, *p* < 0.001) and we consequently decided to focus only on the number of interactions.

Our initial models for the likelihood to interact (binomial mixed model) and the number of interactions (negative binomial mixed model) included the toy ID (e.g., blocks, cellphone, etc.), the sex of the animal and the toy × sex interaction as predictors, to detect sex differences in toy preferences. We then ran a second set of models where the toys were coded according to their gendered categories to explore sex differences in preferences for neutral, masculine, feminine and ambiguous toys. Ultimately, we ran a last set of models where the toys were coded according to their features (hard or plush and zoomorphic or non-zoomorphic) to explore sex differences in the preference for toys’ characteristics.

Analyses also controlled for the side of the cage where the toy was presented, how many trials the animal went preceded the trial in question, as well as the number of times the toy was already seen during the test day. The monkey ID was included as a random factor to functionally nest trials within animals. Final models were generated from backward and forward stepwise selection from the full factorial model and based on Akaike Information Criteria [49]. Where significant interactions indicated sex differences in preferences, we ran post-hoc Tukey tests to identify between-sex and within sex differences and *p* values were adjusted for multiple comparison using the False Discovery Rate method. Effects sizes (Cohen’s *d*) are provided for sex differences in preferences for gendered categories for future inter-studies comparison purposes.

## Results

### Sex differences in the behavior expressed during the test

Males and females did not differ in their likelihood to visit the side compartments containing the toys (Probability ± SE: males = 0.94 ± 0.03, females = 0.93 ± 0.04, *χ*^2^ = 0.10, *p* = 0.76) or to interact with toys overall (Probability ± SE: males = 0.30 ± 0.07, females = 0.35 ± 0.06, *p* = 0.83). When the monkeys interacted with the toys, the number of interactions did not differ between males and females overall (Mean ± SE: males = 5.69 ± 1.5, Mean ± SE: females = 5.45 ± 1.40, *χ*^2^ = 0.08, *p* = 0.90). The likelihood to interact with a toy was not influenced by the number of times the monkeys had seen this toy during the testing day (*p* = 0.24) or by the number of trials they had completed (*p* = 0.73). When animals interacted with toys, the number of interactions with a given toy was not influenced by the number of times this toy was seen during the test day but increased with the accumulated number of trials the monkey had completed (*β* = 0.09 ± 0.04, *χ*^2^ = 5.98, *p* = 0.01). This effect was the same for males and females (sex × trial number: *p* = 0.88).

Males were more likely than females to scratch themselves during the trials (probability ± SE: males = 0.44 ± 0.08, females = 0.22 ± 0.08, *χ*^2^ = 4.52, *p* = 0.03). Females were more likely than males to coo (probability ± SE: males = 0.0008 ± 0.001, females = 0.06 ± 0.07, *χ*^2^ = 4.59, *p* = 0.03).

### Sex differences in toy preferences

The likelihood to interact with a toy depended on the toy itself (*χ*^2^ = 131.09, *p* < 0.001). Sex differences in toy preferences were revealed by a significant interaction, toy × sex (*χ*^2^ = 28.12, *p* = 0.02). Paired comparisons did not reveal any significant difference between males and females in their likelihood to explore any specific toy (all toys: *p* > 0.05). However, males and females showed multiple within-sex (between toys) significant differences in their likelihood to interact with the different toys. Out of the 120 possible toy pair comparisons, 35 were significant in males and 34 in females (following False Discovery Rate *p*-value adjustment). Only 15 of these within-sex comparisons were found in both males and females. These results are detailed in Additional file 1: Table S1 and S2.

**Fig. 2:**
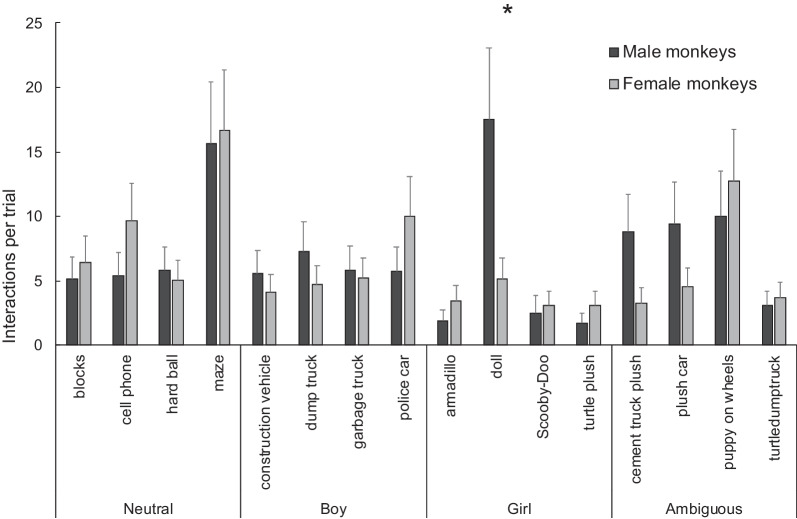
Mean (± SEM) number of interactions with the different toys according to monkeys’ sexes. Our model revealed only one significant between-sex difference: males spent significantly more time interacting with the doll than females. Tukey tests followed by False Discovery Rate *p* adjustment *p < 0.05

When the monkeys interacted with the toys, the number of interactions also depended on the toy itself (*χ*^2^ = 171.04, *p* < 0.001), indicative of population preferences for both males and females. The model also retained the toy × sex interaction (*χ*^2^ = 47.78, *p* < 0.001). Between-sex comparisons revealed a significant difference in the number of interactions for only one of the 16 toys: males interacted more with the doll than females (Mean ± SE: males = 17.50 ± 5.58, females = 5.13 ± 1.62, *t* = − 2.74, *p* = 0.03, Cohen’s *d* = 1.23, Fig. 2).

Males and females showed multiple within-sex (between toys) significant differences in the number of interactions with the different toys. Out of the 120 possible toy pair comparisons, 47 were significant in males and 48 in females (following False Discovery Rate *p*-value adjustment). These results indicate a strong preference of males for the doll and a preference for most toys over the plush armadillo and the plush turtle. Females showed a strong preference for the maze, the cellphone, the police car, and the puppy on wheels over most of the other toys. These results are detailed in Additional file 1: Table S3 and S4.

### Sex differences in preferences for toy gender categories

**Fig. 3:**
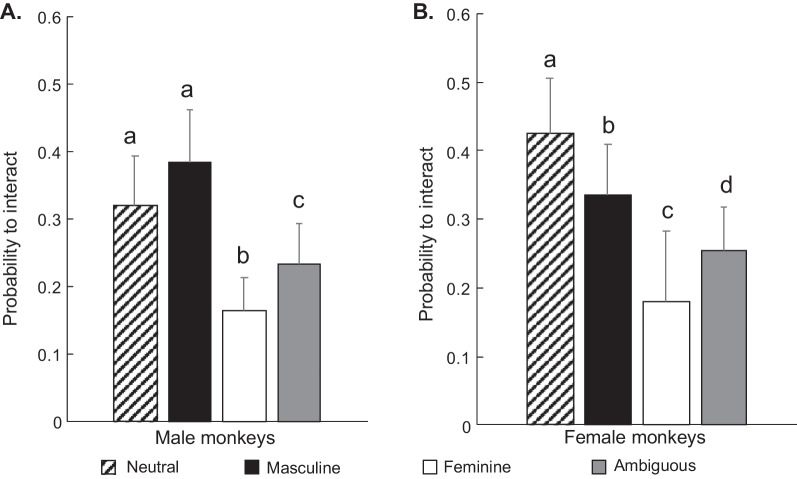
Probability (estimated marginal mean ± SEM) to interact with toys of different gendered categories in males (**A**) and females (**B**). Bars that share a letter do not differ significantly from each other. Bars that do not share a letter differ from each other and denote significant within-sex difference (provided independently for each graph). Tukey tests followed by False Discovery Rate *p* adjustment for multiple comparison

The likelihood to interact with a toy depended on its gendered category (*χ*^2^ = 103.97, *p* < 0.001). Males and females also showed differences in their preferences for toys from different gender categories (gendered category × sex: *χ*^2^ = 9.20, *p* = 0.03). Paired comparisons revealed that males and females did not differ in their likelihood to interact with toys of any gendered category (all ps > 0.05). However, males and females were both more likely to interact with neutral toys than with feminine toys (Cohen’s *d*: *d*_males_ = 0.47; *d*_females_ = 0.69) or ambiguous toys (Cohen’s *d*: *d*_males_ = 0.24; *d*_females_ = 0.44), and with masculine toys more than with feminine (Cohen’s *d*: *d*_males_ = 0.62; *d*_females_ = 0.47) or ambiguous toys (Cohen’s *d*: *d*_males_ = 0.39; *d*_females_ = 0.22) (Fig. 3). Both males and females were also more likely to interact with ambiguous toys than with feminine toys (Cohen’s *d*: *d*_males_ = 0.23; *d*_females_ = 0.24) (Fig. 3). Females, but not males, were more likely to interact with neutral than with masculine toys (Cohen’s *d*: *d*_males_ = 0.14; *d*_females_ = 0.22) (Fig. 3).

**Fig. 4:**
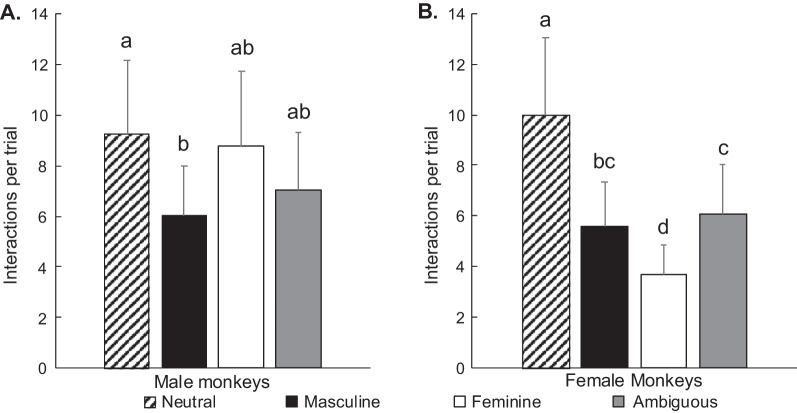
Average number of interactions per trial (estimated marginal mean ± SEM) according to toy gendered categories in males (**A**) and females (**B**). Bars that share a letter do not differ significantly from each other. Bars that do not share a letter differ from each other and denote significant within-sex difference (provided independently for each graph). Tukey tests followed by False Discovery Rate *p*-value adjustment for multiple comparison

When monkeys interacted with the toys, the number of interactions depended on the toy gendered category (*χ*^2^ = 39.02, *p* < 0.001, Fig. 4). These preferences were modulated by sex of the monkey (gendered toy category × sex: *χ*^2^ = 15.85, *p* = 0.001, Fig. 4). Males and females did not differ for their number of interactions with any gender category of toys overall (*p* > 0.05). Within-sex (between gendered categories) comparisons revealed more preferences associated with gendered categories amongst females than amongst males, based on the number of interactions. When they interacted with the toys, males interacted more with neutral toys than with masculine toys (Cohen’s *d* = 0.42) (Fig. 4A). Females interacted more with neutral toys than with masculine (Cohen’s *d* = 0.58), feminine (Cohen’s *d* = 0.98), or ambiguous (Cohen’s *d* = 0.48) toys. Females also interacted more with masculine toys than with feminine toys (Cohen’s *d* = 0.40) and more with ambiguous toys than with feminine toys (Cohen’s *d* = 0.50) (Fig. 4B).

### Sex differences in preferences for toy characteristics (material and shape)

**Fig. 5:**
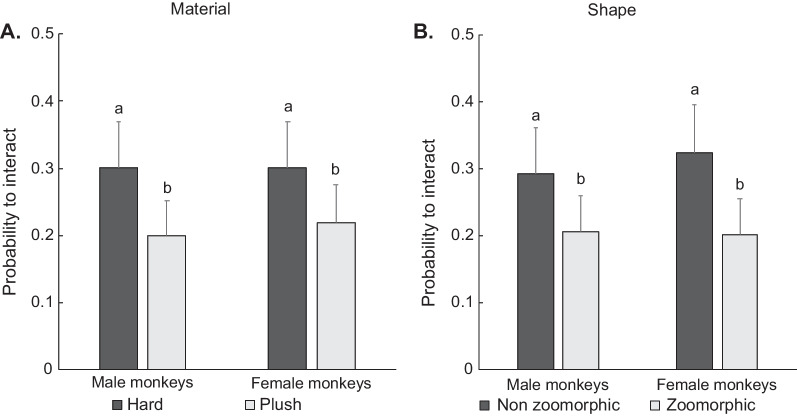
Probability (estimated marginal mean ± SEM) to interact with toys according to toys characteristics. **A** Material, **B** Shape. Males and females showed similar preferences for toys made of hard materials over plush toys and non-zoomorphic over zoomorphic toys. Bars not sharing a letter denote significant difference (provided independently for each graph). Tukey tests followed by False Discovery Rate *p* adjustment for multiple comparisons

Males and females showed similar preferences for toy characteristics, based on the likelihood to interact with the toys. Both males and females were more likely to interact with toys made of hard materials (plastic, metal, or wood) than with plush toys (material: *χ*^2^ = 25.22, *p* < 0.001; Cohen’s *d*: *d*_male_ = 0.48, *d*_female_ = 0.49) and with non-zoomorphic than with zoomorphic toys (shape: *χ*^2^ = 32.38, *p* < 0.001, Chohen’s *d*: *d*_male_ = 0.55, *d*_female_ = 0.55; Fig. 5A, B).

**Fig. 6:**
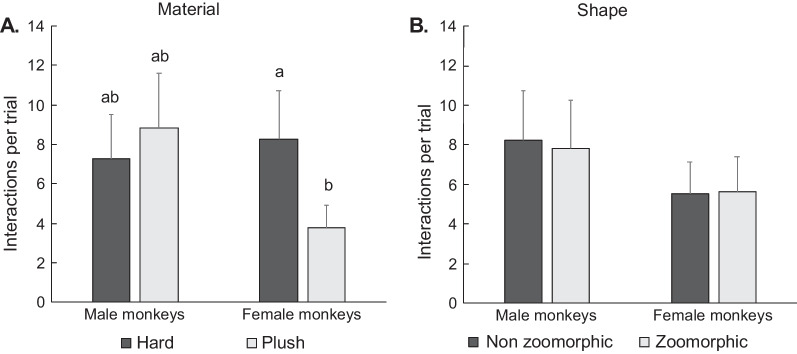
Average number of interactions per trial (estimated marginal mean ± SEM) according to toys characteristics. **A** material, **B** shape. Bars not sharing a letter denote significant difference (provided independently for each graph). Tukey tests followed by False Discovery Rate *p*-value adjustment for multiple comparison

There were, however, sex differences in preferences for toy material based on the number of interactions with the toys (sex × material: *χ*^2^ = 21.84, *p* < 0.001). When they interacted with the toys, males and females did not differ in their interactions according to the shape of the toy, but females interacted more with hard toys than with plush toys (Cohen’s *d* = 0.78) while males did not show a preference (Fig. 6A). Males and females showed no differences in their interactions according to the shape of the toys and neither males nor females expressed preferences according to the shape of the toys (non-zoomorphic v. zoomorphic, Fig. 6B).

## Discussion

We assessed sex differences in monkeys’ toy preferences, as was done previously [9, 10], but using a choice test paradigm with socially isolated animals, a novel experimental approach in this scientific domain. Rhesus monkeys were tested with toys that we assigned to different gendered categories (i.e., “masculine”, “feminine”, “neutral”, “ambiguous”) based on the previous study in rhesus macaques [10]. Overall (considering all trials), male and female monkeys had similar likelihoods of interacting with different types of toys: both sexes preferred neutral and masculine toys, more than ambiguous and feminine toys. Both males and females also had a greater preference (as operationalized by likelihood to interact) for toys made of hard material as compared to plush toys, and for non-zoomorphic over zoomorphic toys.

When they interacted with the toys, males interacted more with the doll than with most toys and interacted more with the doll than the females did. This was the only between-sex difference that emerged in our study. When they interacted with the toys, both males and females interacted more with neutral than with masculine toys (hard vehicles) but while this was the only preference expressed by males related to gendered categories, females showed more preferences and interacted the least with feminine toys. Finally, females interacted more with toys made of hard materials than with plush toy while males did not show preferences for materials. Multiple aspects of our results contrast with the two previous studies in monkeys (vervets: [9], rhesus: [10]). In particular, our results contrast with the previous study in rhesus monkeys [10] that reported male monkeys had preferences for masculine toys and female monkeys had no preference for either masculine or feminine toys.

Unlike the previous studies testing toy preferences in monkeys, we used a choice test in which we presented two toys simultaneously to each subject monkey. Such methods are recommended if the goal is to measure preferences [43] because it makes animals pick between options (rather than quantifying “preference” based on time spent or frequency of interaction with a single object, and then comparing those metrics across objects). Forced choice paradigms like this have been used to test human toy preferences, revealing stronger typical sex differences than other methods [23]. This paradigm is very different from that used by Alexander and Hines [9] who presented only one toy at a time and compared the time vervets spent manipulating each object. However, [10] presented two toys simultaneously as we did on our study. We discuss hereafter multiple possible explanations for why our results do not match the results found in their study and more broadly, do not match the typical pattern of sex differences in toy preferences in humans [11, 23].

**Fig. 7:**
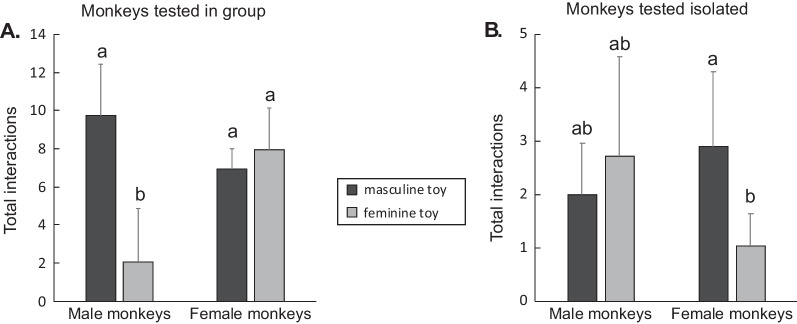
Mean (± SEM) number of interactions with masculine and feminine toys from male and female rhesus monkeys tested in group (**A** adapted from [10] or isolated, **B** the present study). We modified our analysis to match with the study by Hasset et al. [10]: we limited our study to trials offering choices between wheeled (masculine) and plush (feminine) toys only. All our animals generated more than 5 interactions with the toys overall (criteria of data inclusion in the previous study [10]), and were consequently all included in the analysis. Bars that share a letter do not differ significantly from each other. Bars that do not share a letter differ from each other and denote significant difference

Our study evaluated preferences for a larger variety of toys and toys categories than did Hassett et al. [10] and used a slightly different analysis plan to estimate preferences through both the likelihood to interact with the toys and the number of interactions. To make sure discrepancies in the analysis plan and choice of toys did not drive the differences between the studies, we conducted an additional analysis on the average number of interactions for individuals who interacted at least 5 times with toys during the trials presenting only masculine and feminine toys (respectively wheeled and plush toys) mirroring the analysis approach from Hassett et al. [10]. This analysis did not reveal the pattern identified in Hassett et al. [10] (Fig. 7) suggesting that the analysis strategy itself is not the cause of across study differences.

The experimental design of our study followed the toy classification made by Hassett et al. [10], as this is the only study that properly tested preferences of toys in nonhuman primates. We consequently classified wheeled toys as male toys and plush toys as female toys. The classification of the different toys to masculine and feminine categories is not standardized among authors [23]. While vehicles are generally considered as male toys, classification for plush toys is more controversial (e.g., [42]) and past studies were equally likely to classify them as female toys or as neutral toys [23], except plush dolls, typically considered female toys [11, 23]. A wrong a priori classification of toys is at risk of misleading conclusions, but we argue here that our results would not be fundamentally different would we have decided to consider plush toys as neutral toys. Indeed, males were not more likely than females to interact with vehicles and when they did interact with vehicles, they did not interact more than females did. Females were not more likely to interact with the doll than males did, and when they did interact with the doll, they interacted less than males did. Therefore, our results would still contrast with the typical pattern of sex differences in toy preferences in humans would we have decided to classify plush toys, other than the doll, as neutral toys.

Another methodological difference between studies was that in the Hassett et al. [10] study, animals could freely interact with the toys while the toys could not be moved from their initial locations in our study. Toys in our study could be physically manipulated but remained in the small mesh compartment. This could have reduced the ability of animals to generate certain exploratory behaviors and potentially affected our measures of preferences through the number of interactions with the toys. For instance, the propensity of males (both human infants and non-human primates) for vehicle-like toys has been posited as relying on their preference for object that afford propulsive movement [9, 10, 50]. Female monkeys have been proposed to show preferences for objects that resemble features of monkey infants [51] due to their greater interest in younger infants [52]. In the wild, young chimpanzee females tended to carry sticks in a way that suggests a rudimentary doll play more often than males did [53]. In this view, sex differences may not have emerged in our study because while monkeys could touch and manipulate the toys, they could not propel them through space or cradle them. This idea is called into question by the fact that studies in humans have demonstrated that the emergence of sex differences in toys does not require the motor activity associated with the toy [31]. Further, differences in the ability to propel objects does not explain why the most potent sex difference that we identified was that males manipulated the doll more than females.

For a variety of reasons, we also believe that it is unlikely that the limited access to the toys affected likelihood of interacting at all with the toys—which was our measure of preferences. Whether or not the monkeys interacted at all with the toys should be contingent more on the visual attractiveness of the different toys than their ability to propel them (which would have a greater impact on longer lasting manipulation behaviors). This index revealed no preference of males for vehicles and no preference of females for plush toys (or for the doll, with a more restrictive categorization) in our monkeys. This result is inconsistent with results found in human infants showing visual preference for sex-congruent toys as early as 3–8 months old [31]. While infants at this age are posited to not seek conformism with external referents, they are nevertheless exposed during infancy to gender congruent toys which might have an experience-dependent effect on their visual preferences [31, 54]. Therefore, it is possible that the visual preference for sex-congruent toys in humans relies more on such socially induced familiarity with specific toys than on biologically rooted sex differences in visual preferences for toys characteristics. Supporting this hypothesis, when 1 year old children are tested for visual preference for shape or texture rather than for specific toys, they do not show sex differences [55]. Similarly, we did not find sex differences in the likelihood to interact with the toys based on toys characteristics, consistently with previous work in nonhuman primates [51].

We propose that the emergence of human-typical sex differences in nonhuman primate’s toy preferences depend on the social context in which interactions with toys is observed. The human sex difference pattern [11], previously observed in nonhuman primates (rhesus: Hassett et al. [10],vervets: Alexander and Hines [9]) emerged when group housed monkeys were tested in social groups. In contrast, our group-reared, pair-housed monkeys were tested alone and did not generate this pattern of behavior. This could have impacted preferences by influencing who can access the toys (e.g., a particular type of toy could be monopolized by some members of the group) or because individuals from one sex are less likely to compete for it. Dominance status, for instance, is likely to influence access to the different toys. In the previous study of rhesus, higher ranking monkeys interacted more with toys and the females with no toy preferences were the lowest ranking [10]. Similarly, higher ranking vervets, regardless of sex, tended to interact more with masculine and less with feminine toys than lower ranking individuals [9]. Based on how we tested animals, the group dynamics that impacted the results of the previous studies were absent in our study.

Access to toys is not the only reason that group testing might influence behavioral outcomes. Another possibility is that the group social context is necessary for the emergence of human-typical sex differences in toy preferences because males and females do not differ in their individual preferences for the toys but differ in their preferences for the social activities that are or could be facilitated by the toys. Toy preferences have been proposed to parallel these social activity differences in humans [56] but also in nonhuman primates. According to this hypothesis, male monkeys prefer objects allowing for propulsion due to their generally more social active playstyle [57–59]. Therefore, preferences are not observed if the social activity cannot be expressed.

Finally, the social environment of our monkeys could have impacted our results. The monkeys in the previous studies lived and were tested in large social groups, which can create conditions in which the social behavior of males and females is fairly different. The dynamics of rich social groups are said to maintain such social differences while sex differences are reduced when animals are housed in mixed sex dyads [60]. Therefore, the social conditions in which the animals in the present study were housed may have contributed to our effects.

On a final note, our observations highlight another novel result: the only between-sex difference we observed is that, when males interacted with the doll, they interacted more with it than females did. One argument is that a lack of interest of the females for the doll could be a result of their reproductive history since our females had infants in the past which could have led to a decreased interest in play-mothering activities [53]. However, females and males were equally likely to interact with the doll, suggesting a similar visual interest. Additionally, most of the monkeys included in the two previous studies were old enough to be parous and authors did not report any effect of age or parity on toy preferences [9, 10]. The higher number of interactions of males with the doll is a new result that needs to be replicated by further studies in nonhuman primates assessing toy preferences in different social conditions.

### Perspective and significance

Our study found that males and females rhesus monkeys tested alone did not show the same pattern of toy preferences as young male and female children or as monkeys in previous experiments. This result calls into question the existence of a strong biological basis supporting sex differences in toy/object preferences in humans. The existence of a strong biological basis to sex differences in toy preferences in humans is mainly supported by visual preferences for gender congruent toys at a very young age [31] and preference for masculine toys in young girls with CAH [29]. However, these latter findings can also be largely driven by social factors. Young boys and girls can develop differences in visual preferences at a very young age due to divergences in the environments that parents design for their sons and daughters [55]. Similarly, young girls with CAH preferences for boy toys could be induced by their preference for playstyles preferred by males and for male playmates, which could also socially induce their preference for masculine toys. Further investigation is required to better understand how the past and current social environment influence the expression of sex differences in primates, including humans.

## Conclusion

In conclusion, rhesus monkeys (*Macaca mulatta*) express preferences for different objects and these preferences depend on the sex of the individual and the social context. The most robust sex difference to emerge when monkeys interacted with the doll, male monkeys did so much more frequently than female monkeys—standing in stark contrast to previous reports. These results are of major importance for future experimental designs in behavioral neuroscience where the measure of interactions with objects are central to many experimental designs. In such experiments, and despite the primate exemption to include sex as a biological variable in primates, it appears critical to account for the sex of tested animals in the design, analysis and translational conclusions drawn from our studies.

## Supplementary Information

**Additional file 1: Table S1.** Males’ toys preferences based on likelihood to interact. If significant difference is detected, preferred toy is mentioned in the cell. Tukey tests followed by False Discovery Rate P adjustment. **Table S2.** Females’ toys preferences based on likelihood to interact. If significant difference is detected, preferred toy is mentioned in the cell. Tukey tests followed by False Discovery Rate P adjustment. **Table S3.** Males’ toys preferences based on the number of interactions per trial when they interacted with the toy. If significant difference is detected, preferred toy is mentioned in the cell. Tukey tests followed by False Discovery Rate P adjustment. **Table S4.** Females’ toys preferences based on the number of interactions per trial when they interacted with the toy. If significant difference is detected, preferred toy is mentioned in the cell. Tukey tests followed by False Discovery Rate P adjustment.

## Data Availability

Data will be posted publicly on osf.io upon acceptance of the publication. If editor would like the data as part of review, please let us know.

## Footnotes

1 We acknowledge the complexity of gender constructs in humans [19] which are far more complex than simply “male” and “female”. Given that we are working with monkeys, and it is unclear whether they have gender identity, we rely on a sex dichotomy for this paper discussing “male” and “female”, “boy” and “girl”.
