# Supplementary material for: Monkeys do not show sex differences in toy preferences through their individual choices
Source: Biol Sex Differ. 2023 Feb 3;14:3. doi: 10.1186/s13293-023-00489-9 (PMC9898904; doi:10.1186/s13293-023-00489-9)
Supplement: Supplementary file 1 — Additional file 1: Table S1. Males’ toys preferences based on likelihood to interact. If significant difference is detected, preferred toy is mentioned in the cell. Tukey tests followed by False Discovery Rate P adjustment. Table S2. Females’ toys preferences based on likelihood to interact. If significant difference is detected, preferred toy is mentioned in the cell. Tukey tests followed by False Discovery Rate P adjustment. Table S3. Males’ toys preferences based on the number of interactions per trial when they interacted with the toy. If significant difference is detected, preferred toy is mentioned in the cell. Tukey tests followed by False Discovery Rate P adjustment. Table S4. Females’ toys preferences based on the number of interactions per trial when they interacted with the toy. If significant difference is detected, preferred toy is mentioned in the cell. Tukey tests followed by False Discovery Rate P adjustment. [file 13293_2023_489_MOESM1_ESM.pdf]

Table S1. **Males' toys preferences based on likelihood to interact**. If significant difference is detected, preferred toy is mentioned in the cell. Tukey tests followed by False Discovery Rate P adjustment.

**Neutral**	blocks																
	cellphone																
	hard ball																
	maze																
**Boy**	construction vehicle																
	dump truck																
	garbage truck																
	police car																
**Girl**	armadillo			**hardball**	**maze**	**construction vehicle**	**dump truck**	**garbage truck**	**police car**								
	doll									**doll**							
	scoobydoo	**blocks**	**cellphone**	**hardball**	**maze**	**construction vehicle**	**dump truck**	**garbage truck**	**police car**		**doll**						
	turtle plush			**hardball**	**maze**	**construction vehicle**	**dump truck**	**garbage truck**	**police car**								
**Ambiguous**	cement truck plush						**dump truck**					**cement truck plush**					
	plush car				**maze**	**construction vehicle**	**dump truck**	**garbage truck**				**plush car**					
	puppy on wheels				**maze**	**construction vehicle**	**dump truck**	**garbage truck**				**puppy on wheels**					
	turtle dump truck											**turtle dump truck**					
		blocks	cellphone	hard ball	maze	construction vehicle	dump truck	garbage truck	police car	armadillo	doll	scoobydoo	turtle plush	cement truck plush	plush car	puppy on wheels	turtle dump truck
		**Neutral**				**Boy**				**Girl**				**Ambiguous**			

Table S2. **Females' toys preferences based on likelihood to interact**. If significant difference is detected, preferred toy is mentioned in the cell. Tukey tests followed by False Discovery Rate P adjustment.

**Neutral**	blocks																
	cellphone																
	hard ball																
	maze	**maze**		**maze**													
**Boy**	construction vehicle				**maze**												
	dump truck				**maze**												
	garbage truck																
	police car				**maze**												
**Girl**	armadillo		**cellphone**	**hard ball**	**maze**			**garbage truck**									
	doll		**cellphone**		**maze**			**garbage truck**									
	scoobydoo		**cellphone**	**hard ball**	**maze**			**garbage truck**									
	turtle plush	**blocks**	**cellphone**	**hard ball**	**maze**	**construction vehicle**		**gardage truck**	**police car**								
**Ambiguous**	cement truck plush		**cellphone**		**maze**			**garbage truck**									
	plush car				**maze**								**plush car**				
	puppy on wheels		**cellphone**		**maze**			**garbage truck**									
	turtle dump truck		**cellphone**		**maze**			**garbage truck**									
		blocks	cellphone	hard ball	maze	construction vehicle	dump truck	garbage truck	police car	armadillo	doll	scoobydoo	turtle plush	cement truck plush	plush car	puppy on wheels	turtle dump truck
		**Neutral**				**Boy**				**Girl**				**Ambiguous**			

Table S3. **Males’ toys preferences based on the number of interactions** per trial when they interacted with the toy. If significant difference is detected, preferred toy is mentioned in the cell. Tukey tests followed by False Discovery Rate P adjustment.

**Neutral**	blocks																
	cellphone																
	hard ball																
	maze	**maze**	**maze**	**maze**													
**Boy**	construction vehicle				**maze**												
	dump truck				**maze**												
	garbage truck				**maze**												
	police car				**maze**												
**Girl**	armadillo		**cellphone**	**hard ball**	**maze**	**Construct. vehicle**	**dump truck**	**garbage truck**	**police car**								
	doll	**doll**	**doll**	**doll**		**doll**	**doll**	**doll**	**doll**	**doll**							
	scoobydoo				**maze**						**doll**						
	turtle plush	**blocks**	**cellphone**	**hard ball**	**maze**	**construction vehicle**	**dump truck**	**garbage truck**	**police car**		**doll**						
**Ambiguous**	cement truck plush									**cement truck plush**	**doll**		**cement truck plush**				
	plush car									**plush car**			**plush car**				
	puppy on wheels									**puppy on wheels**		**puppy on wheels**	**puppy on wheels**				
	turtle dump truck				**maze**		**dump truck**				**doll**			**cement truck plush**	**plush car**	**puppy on wheels**	
		blocks	cellphone	hard ball	maze	construction vehicle	dump truck	garbage truck	police car	armadillo	doll	scoobydoo	turtle plush	cement truck plush	plush car	puppy on wheels	turtle dump truck
		**Neutral**				**Boy**				**Girl**				**Ambiguous**			

Table S4. **Females' toys preferences based on the number of interactions** per trial when they interacted with the toy. If significant difference is detected, preferred toy is mentioned in the cell. Tukey tests followed by False Discovery Rate P adjustment.

		Neutral				Boy				Girl				Ambiguous			
		blocks	cellphone	hard ball	Maze	construction vehicle	dump truck	garbage truck	police car	armadillo	doll	scoobydoo	turtle plush	cement truck plush	plush car	puppy on wheels	turtle dump truck
**Neutral**	blocks																
	cellphone																
	hard ball		**cellphone**														
	maze	**maze**	**maze**	**maze**													
**Boy**	construction vehicle		**cellphone**		**maze**												
	dump truck		**cellphone**		**maze**												
	garbage truck		**cellphone**		**maze**												
	police car			**police car**		**police car**	**police car**	**police car**									
**Girl**	armadillo		**cellphone**		**maze**				**police car**								
	doll		**cellphone**		**maze**				**police car**								
	scoobydoo	**blocks**	**cellphone**		**maze**				**police car**								
	turtle plush		**cellphone**		**maze**				**police car**								
**Ambiguous**	cement truck plush		**cellphone**		**maze**				**police car**								
	plush car		**cellphone**		**maze**				**police car**								
	puppy on wheels	**puppy on wheels**		**puppy on wheels**		**puppy on wheels**	**puppy on wheels**	**puppy on wheels**		**puppy on wheels**	**puppy on wheels**	**puppy on wheels**	**puppy on wheels**	**puppy on wheels**	**puppy on wheels**		
	turtle dump truck		**cellphone**		**maze**				**police car**							**puppy on wheels**	
